# The Value of Interdisciplinary Collaboration in Lateral Medullary Syndrome Rehabilitation: A Case Report

**DOI:** 10.7759/cureus.40065

**Published:** 2023-06-06

**Authors:** Andrew B Herson, Justin D Falk, Davong D Phrathep, Chigozie B Igbonagwam, Steven T Fischer, Brooke T Miller, Daniel Leary

**Affiliations:** 1 Physical Medicine and Rehabilitation, Lake Erie College of Osteopathic Medicine, Jacksonville, USA; 2 Physical Medicine and Rehabilitation, AdventHealth Tampa, Tampa, USA

**Keywords:** cerebrovascular accident (stroke), speech and swallowing therapy, occupational therapy, physical therapy rehabilitation, rehab, lateral medullary syndrome (wallenberg syndrome), wallenberg’s syndrome

## Abstract

Wallenberg’s syndrome, also known as lateral medullary syndrome (LMS), is a neurological condition resulting from damage to the lateral portion of the medulla oblongata. We present a case of a 64-year-old man with Wallenberg’s syndrome who presented for acute rehabilitation after sustaining a cerebrovascular accident (CVA). As seen in our patient, common symptoms of LMS include difficulty swallowing, hoarseness, ipsilateral weakness, and ipsilateral loss of sensation or numbness. Although the prognosis following infarction is often good, dysfunction in swallowing is one of the key deficits that have a long-term impact on patient quality of life. We aim to emphasize the significance of the interdisciplinary approach to achieving favorable health outcomes in patients with LMS.

## Introduction

Wallenberg’s syndrome, also called lateral medullary syndrome (LMS), is a neurological disorder associated with symptoms that result from vascular injury to the lateral medulla oblongata. This primarily occurs due to occlusion of the vertebral or posterior inferior cerebellar artery (PICA), along with cavernous angiomas and malignancies [1,2]. As the most prevalent posterior ischemic stroke syndrome, clinicians estimate that there are more than 60,000 new cases each year in the United States [3]. Risk factors for LMS are dependent on age but generally include atherosclerosis, coronary artery disease, hypertension, and diabetes [1].

The presentation of LMS is variable depending on the extent of damage to the lateral medulla. The most common symptoms are dysphagia, ataxia, vertigo, gaze abnormalities, nausea, vomiting, reduced ipsilateral pain and temperature sensation, hypoventilation syndrome, and contralateral body weakness [1]. The diagnosis of LMS is usually suspected from clinical examination and history of presentation [4]. The most sensitive diagnostic test to confirm infarction in the inferior cerebellar area or lateral medulla is magnetic resonance imaging (MRI) with diffusion-weighted imaging (DWI) [5].

Similarly, to other forms of ischemic stroke, LMS can benefit from thrombolytics when administered within 4.5 hours of the onset of symptoms [1]. Stroke syndromes like LMS can cause permanent disability and affect a patient’s normal functional level. Here we present a case of LMS in a 64-year-old man highlighting the importance of the interdisciplinary approach to achieve successful health outcomes related to symptom management and rehabilitation of patients with LMS.

## Case presentation

A 64-year-old man with a history of diabetes mellitus and hypertension was admitted to our hospital for acute rehabilitation after a cerebrovascular accident (CVA). He had previously sought treatment at another hospital due to left facial numbness. On presentation at the other hospital, he had a blood pressure of 216/91 mmHg, pulse rate of 87 bpm, temperature of 97.5 °F (36.4 °C) (oral), respiratory rate of 18, and an Sp0_2_ of 98%.

On physical exam, the patient had slight left-sided facial droop and numbness, a hoarse-sounding voice, scattered rhonchi, and ataxia. Laboratory tests included an activated partial thromboplastin time (aPTT) test, a urinalysis, a complete blood count (CBC) with differential, and a basic metabolic panel (BMP). The urinalysis was normal. The CBC with differential revealed normocytic normochromic anemia, neutrophilia, lymphocytopenia, and a low aPTT (Table 1).

**Table 1:** Complete Blood Count with Differential aPTT, activated partial thromboplastin time; L, liters; g/dL, grams per deciliter; μm^3^, cubic micrometers; mm^3^, cubic millimeters; fL, femtoliters; Hb/cell, hemoglobin per cell

Marker	Laboratory values	Reference range
White blood cell count	9.04	4.5-11.0 x 10^9^/L
Red blood cell count	4.74	4.3-5.9 x 10^12^/L
Hemoglobin	13.7	13.5-17.5 g/dl
Hematocrit	39	41.0-53.0%
Mean corpuscular volume	83	80-100 μm^3^
Mean corpuscular hemoglobin	29	27-34 g/dl
Mean corpuscular hemoglobin concentration	35	32-36% Hb/cell
Red blood cell distribution width	12.9	11.7-14.4%
Platelet count	202,000	150,000-400,000 mm^3^
Mean platelet volume	11.9	7.2-12.3 fL
Immature granulocyte %	0	0.0-1.0%
Neutrophil %	79	45-75%
Lymphocyte %	15	20-45%
Monocyte %	6	3.5-9.0%
Eosinophil %	0	0-8%
Basophil %	0	0-2%
aPTT	21.4	25-40 seconds

The BMP revealed hyponatremia, hypermagnesemia, and significant hyperglycemia (Table 2).

**Table 2:** Basic Metabolic Panel BUN, blood urea nitrogen; eGFR, estimated glomerular filtration rate; mg/dL, milligrams per deciliter; mL/min/1.73m^2^, milliliters per minute per 1.73 meters squared; mmol/L, millimoles per liter

Marker	Laboratory value	Reference range
Sodium	131	135-145 mmol/L
Potassium	3.9	3.5-5 mmol/L
Chloride	95	95-105 mmol/L
Carbon dioxide	25	22-32 mmol/L
Anion gap	11	4 to 12 mmol/L
BUN	20	7-25 mg/dL
Creatinine	1.19	0.7-1.5 mg/dL
Glucose	562	70-100 mg/dL
Calcium	9.8	8.5-10.5 mg/dL
Magnesium	2.2	0.75-1.0 mmol/L
eGFR	68	>60 mL/min/1.73m^2^

 

A computed tomography angiogram (CTA) showed occlusion of the left vertebral artery and 75% stenosis of the distal right vertebral artery, leading to left LMS (Wallenberg’s syndrome) with associated hiccups, vocal cord paralysis, mild left eye ptosis, dysphagia, and dysmetria. Left lateral medullary stroke was confirmed by MRI. The patient was not a candidate for thrombolysis or thrombectomy due to uncertainty about time from the patient’s last known normal as well as his anticoagulation medication history.

During his hospital stay, the patient developed complications such as hyperosmolar hyperglycemic state, hypertensive urgency, aspiration pneumonia, metabolic alkalosis, and dysphagia. He received IV antibiotics for pneumonia, percutaneous endoscopic gastrostomy (PEG) placement, and ENT consultation confirmed left vocal cord paralysis. After being deemed medically stable, he was transferred to our hospital for acute rehabilitation.

Upon presentation to our institution, initial vital signs were within normal limits. Imaging included a modified barium swallow (MBS), a chest X-ray, and an esophagogastroduodenoscopy (EGD). On physical exam, the patient had a left-sided PEG tube, slight left-sided facial droop and numbness, a hoarse-sounding voice, bilateral scattered rhonchi, and showed ataxia.

Prior to his CVA, the patient was independent with all mobility and activities of daily living (ADLs) without assistive devices. He developed impairment of mobility, impairment of ADLs, impaired balance, and was deconditioned. After review by physical therapy (PT), speech therapy (ST), and occupational therapy (OT), the patient was deemed to have good rehabilitation potential. He had a good support system at home, and his level of function was addressed, including self-care and mobility (Table 3).

**Table 3:** Current Level of Function Minimal assistance, OT provides 1-25% effort and patient provides 75-99% effort; moderate assistance, OT provides 26-50% effort and patient provides 50-74% effort; maximal assistance, OT provides 51-75% effort and patient provides 25-49% effort; total assistance: OT provides 76-100% effort and patient provides 0-24% effort; ft, feet; rw, rolling walker; gb, gait belt; vc, verbal cue; lob, loss of balance; eob, end of bed; OT: occupational therapy

Activities of daily living	Level of assistance
Eating/feeding assist	Total assistance
Oral hygiene assist	Moderate assistance
Toileting assist	Maximal assistance
Shower/bathe assist	Maximal assistance
Upper body dressing assist	Maximal assistance
Lower body dressing assist	Maximal assistance
Roll right and left assist	Maximal assistance
Sit to lying assist	Maximal assistance
Lying to sit eob assist	Maximal assistance
Sit to stand assist	Maximal assistance
Bed/chair transfer assist	Maximal assistance
Toilet transfer assist	Maximal assistance
Ambulation distance	10 ft, rw, gb, vc, lob, decreased cadence

The patient participated in PT, ST, and OT. PT involved ambulation training with appropriate assistive devices, with a focus on the right lower extremity weakness, progressive mobilization, and safe transfers. ST involved swallow training, oral hygiene education, and electrical muscle stimulation (ESTIM). OT involved ADL training and focus on the patient's right upper extremity weakness. We also involved rehabilitation recreation therapy for diversion and leisure activities and community reentry preparation. Social services was involved in discharge planning and to address the procurement of a two-wheeled walker.

**Figure 1:**
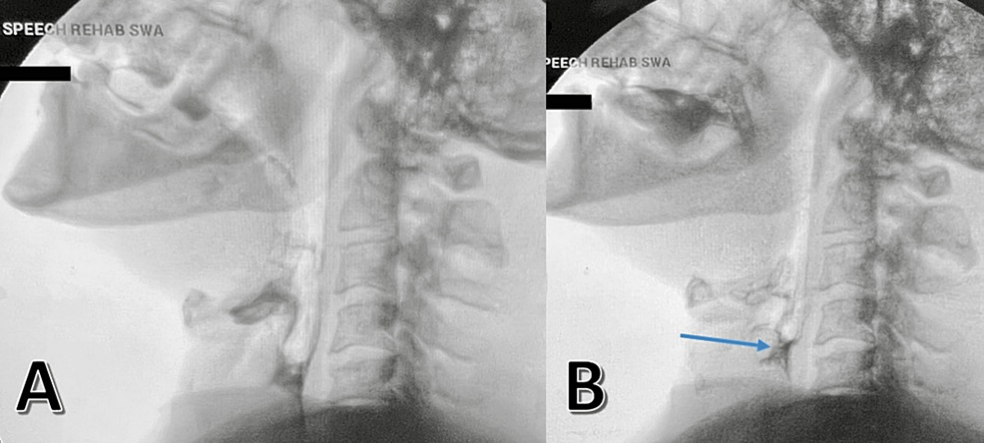
Modified Barium Swallow Modified barium swallow images showing poor to no posterior pharyngeal wall motion and residual pools in the back of the throat (blue arrow).

Due to the patient’s history of non-compliance, vocal cord paralysis, and dysphagia, he was placed on strict NPO and was cleared for ice chips only after good oral hygiene. Respiratory sputum culture was obtained that showed moderate normal respiratory flora. An MBS was performed (Figure 1).

For the patient’s dysphagia, ST performed ESTIM with good results and noted that vocal cord paralysis was not the patient’s main deficit. The patient was instructed to swallow five times for every bolus of food or liquid. He was educated on the importance of making sure he completed the five swallows as it would help lower his chance of aspiration. 

Throughout the patient’s stay, he required consults from multiple specialties for complaints of abdominal distention, sinus pressure and congestion, excessive oral secretions, and increased gastric reflux. The patient had multiple large bowel movements after treatment with MiraLAX but had worsening abdominal distention and continued intolerance of tube feeds. After checking residuals on two occasions, 250 mL were removed. When flushing the patient's PEG tube for medications, large amounts of dark brown fluid were aspirated. Tube feeds were stopped. Gastroenterology was consulted, and it was found that the feed formula was too thick. Tube feeds were restarted with a thinner, lower fiber formula. Erythromycin and Reglan 10 mg IV q.6 were started to increase stomach emptying. The patient began to tolerate the feeds well. The patient also complained of sinus pressure and congestion. There was left-sided maxillary sinus tenderness and left ear pain/fullness. For this, cetirizine was started. Though it helped the initial complaints, the patient continued to have copious amounts of oral secretions. After collaboration with gastroenterology and otolaryngology, the cetirizine was switched to a trial of a scopolamine patch. A Protonix drip was started for the patient’s complaints of gastric reflux. An EGD was ordered, revealing gastric mucosa with focal ulceration, acute inflammation, and focal intestinal metaplasia, consistent with Barrett’s esophagus of the distal esophagus.

The patient showed high motivation and made great strides in his physical rehabilitation. He elected to go to the gym for therapy and tried to start transfers before the chair was in place. He progressed from not being able to walk to being able to walk 10 feet with the assistance of a walker, eventually being able to walk 30+ feet with a walker. His speech got stronger, and he became more understanding of his new physical limitations. Throughout his stay, the patient required intermittent catheterization and failed a voiding trial. He was discharged with a Foley catheter, tube feeds, and a two-wheeled walker.

## Discussion

Wallenberg’s syndrome is the most prevalent posterior ischemic stroke syndrome [6]. About 800,000 Americans suffer from acute stroke each year and 83% of strokes are ischemic in etiology [6]. A total of 20% of ischemic strokes occur in the posterior circulation [6]. Clinicians estimate that there are more than 60,000 new cases of Wallenberg’s syndrome each year in the United States [6]. Infarcts occur more frequently in individuals who consume alcohol [7]. 75% of cases are attributed to large artery atherothrombosis, followed by cardio-embolism at 17% and vertebral dissection at 8% [6]. Other etiologies include hypoplastic vertebral artery, Moya-Moya disease, and vertebrobasilar dolichoectasia [7]. Less common occurrences are seen in individuals with predisposing conditions including subclavian steal syndrome, Fabry disease, and mitochondrial encephalopathy [7]. The most frequently involved vessels are the PICA and the vertebral artery [8].

The vertebral artery has four segments, with the fourth segment being the largest. The PICA originates from the fourth segment of the vertebral artery that arises from the subclavian artery [6]. The PICA has five segments: anterior medullary, lateral medullary, tonsillomedullary, telovelotonsillar, and cortical [6]. The lateral medullary segment extends near the origin of the glossopharyngeal, vagus, and accessory nerve roots [6]. PICA supplies the medulla, the choroid plexus, the tela choroidea of the fourth ventricle, the cerebellar tonsils, the inferior vermis, and the lower aspect of the cerebellar hemisphere. The PICA provides a major contribution to the blood supply of the choroid plexus and the majority of branches of the choroid plexus [6].

Anatomically, the PICA is the artery of interest in Wallenberg’s syndrome; however, studies have shown that the vertebral artery is the most responsible artery [8]. If occluded, PICA circulation can impact CN V, IX, X, and XI in addition to the vestibular nucleus and the nucleus ambiguus. The manifestation of Wallenberg’s syndrome varies depending on the exact location of the damage. Vertigo, nystagmus, nausea, and vomiting occur with the involvement of the vestibular nucleus [8]. Dysphagia, dysphonia, and dysarthria occur with damage to the nucleus ambiguus, glossopharyngeal, and vagus nerves [8]. These symptoms can sometimes include ipsilateral loss of gag reflex and hiccups [8]. Dysphagia is more severe in patients with Wallenberg’s syndrome [7]. Ipsilateral ataxia occurs with damage to the cerebellum and spinocerebellar fibers [8]. Ipsilateral Horner syndromes occur with damage to the sympathetic fibers. Impairment of pain and temperature sensation to the ipsilateral face and contralateral trunk and limbs occur with damage to the spinal trigeminal nucleus or spinothalamic tracts. Patients with Wallenberg’s syndrome may have any combination of symptoms. Sensory disturbances are the most frequent manifestation of symptoms and are seen in 90% of patients [8].

In the acute setting, it is recommended all patients that exhibit stroke symptoms be initially evaluated with non-contrast CT. In the setting of ischemic injury, follow-up studies such as CT angiography, CT-perfusion imaging, and MRI with DWI are conducted [9]. The best diagnostic test to confirm infarction in the inferior cerebellar area or lateral medulla is MRI with DWI [5]. Additionally, CT or magnetic resonance angiogram can help identify the site of vascular occlusion and rule out other etiologies, like vertebral artery dissection [10]. In the setting of cardiac pathologies, an electrocardiogram (ECG), echocardiogram, and serum electrolytes can be used to exclude underlying arrhythmias, including atrial fibrillation, and left atrial enlargement that may be indicative of cardioembolic origin [6].

Once an acute ischemic stroke has been confirmed, it is necessary to determine the patient’s time from their last known normal. This will determine the potential acute treatment options [9]. In addition, most comprehensive stroke centers will use the Alberta stroke program early CT score (ASPECTS), or posterior circulation ASPECTS (pc-ASPECTS), to determine the likelihood of tissue being recovered after an ischemic injury [11]. If it is within the first 4.5 hours of the last known normal, patients qualify for thrombolytic therapy. A mechanical thrombectomy is an option for treatment given patients meet adequate clinical criteria. Posterior circulation mechanical thrombectomies are not common and not well studied given the novelty of this field; however, current studies seem to suggest a similar benefit to posterior circulation thrombectomies as seen in anterior circulation [12].

As discussed previously, there are a myriad of impairments that can occur after infarction to the lateral medulla. The most notable is dysphagia. In patients who have significant dysphagia, prior studies have shown there is a benefit of swallowing training to reduce aspiration incidences [13]. Unfortunately, there are many patients who do require a PEG placed indefinitely, even with swallowing training. Another symptom related to this syndrome that substantially impacts the quality of life is trigeminal neuralgia. This is a syndrome related to many other traumatic injuries to the brain but is significant given its long-term impact on the patient. Medications such as carbamazepine or ox carbamazepine can be used, but surgical options are also possible [14]. Overall, the prognosis for LMS is dependent on the degree of disability after infarction. In some cases, patients can make a full recovery [13].

According to recent studies, the interdisciplinary team has been shown to be the best in stroke, acquired brain injury, back pain, mental health, and chronic pain [15]. Evidence from published scientific literature from larger trials indicates physical medicine and rehabilitation programs with multidisciplinary teams achieve better results [16]. Good teamwork may have a significant influence on survival and, therefore, multidisciplinary training allows specific expertise from each team member to diagnose and assess the severity of health problems. We present a patient who participated in PT, ST, and OT with focuses on assistive devices for the right upper and lower extremity, mobilization, safe transfers, and return to recreational activities and community involvement. Additionally, our patient was involved in the procurement of a two-wheeled walker through social services to help facilitate the home health and community integration process regarding ambulation. Due to the patient’s history of non-compliance, our case highlights the important nature of interactions between rehabilitation team members and the patient, especially when working toward common patient goals. At the beginning of the rehabilitation process, our patient demonstrated vocal cord paralysis and dysphagia. He was placed on strict NPO and was cleared for ice chips only after good oral hygiene. With time, the patient improved in the rehabilitation process by expressing intelligible words with the help of ST. Additionally, with the help of PT and OT, he went from not being able to walk to walking 30 feet with a walker and being moderate assistance for transfers now. Our case demonstrates an intentional holistic view of our patient’s plan of care, and as a prerequisite for safe intervention, we used a multidisciplinary approach for the improvement of our patient’s condition.

## Conclusions

Effectiveness is higher in teams working with the interdisciplinary approach. With the increased complexity of certain conditions, like LMS, the interdisciplinary collaboration between physical medicine and rehabilitation, neurology, and neurosurgery is an important factor in improving health outcomes in patients. Although the patient has shown significant improvements so far, we continually encourage his participation and continued commitment to the success of his rehabilitation journey. Here we emphasize how a rehabilitation treatment plan for patients with LMS must incorporate a multidisciplinary approach to develop, evaluate, and execute treatment based on the goals of every patient.
